# Introducing a New Technique for Fascial Closure to Avoid Renal Allograft Compartment Syndrome in Pediatric Recipients: The Use of Tutoplast^®^ Fascia Lata

**DOI:** 10.3389/fsurg.2022.840055

**Published:** 2022-05-06

**Authors:** Beatriz Bañuelos Marco, Berenice Bergel, Tamara Geppert, Dominik Müller, Anja Lingnau

**Affiliations:** ^1^Department Paediatric Urology, Charité Universitätsmedizin Berlin, Berlin, Germany; ^2^Charité Universitätsmedizin Berlin, Berlin, Germany; ^3^Department of Paediatric Nephrology, Charité Universitätsmedizin Berlin, Berlin, Germany

**Keywords:** Tutoplast^®^, RACS, pediatric kidney transplantation, abdominal compartment syndrome, en bloc, living donor, cadaver donor

## Abstract

**Introduction:**

Renal allograft compartment syndrome (RACS) is a complication that infrequently occurs after renal transplantation. Tight muscle closure may lead to RACS due to compression of renal parenchyma or kinking of the renal vessels. Many techniques have been proposed in an attempt to achieve tension-free closure, which can be specially challenging in child recipients. We present our experience with Tutoplast^®^ Fascia Lata (RTI Surgical Tutogen Medical GmbH Industriestrasse 6, 91077 Neunkirchen am Brand, Germany) closure.

**Methods:**

All pediatric patients who underwent renal transplantation in our center between 2012 and 2021 were reviewed. Eight patients with Tutoplast^®^ Fascia Lata placed at the time of initial transplantation were identified. Donor and recipient characteristics, Doppler ultrasound findings, and overall patient and graft survival rates were analyzed.

**Results:**

Doppler ultrasound was performed intra-operatively after abdominal wall closure. If any sign of vascular compromise was seen, the abdominal wall was opened and the graft was revised. The Tutoplast^®^ Fascia Lata implant was used to perform tension-free fascia closure and, afterwards, a Doppler ultrasound was performed to confirm the optimal renal artery perfusion and venous patency. Three of the renal transplantations were from a cadaver donor, with two of them en bloc. Living donor transplantation was performed in four cases. Among which, there was a case of auto-transplantation due to bilateral renal artery stenosis. None of the patients presented any complications of either short or long term that was derived from the abdominal closure with Tutoplast^®^ Fascia Lata. There was also no record of graft failure till datum.

**Conclusions:**

Restricted volume of the recipient pelvic cavity and the size discrepancy between the recipient pelvic cavity space and the donor adult kidney may lead to RACS. Other situations that occur more infrequently, i.e., as en bloc or auto-transplantation, are prone to suffer the same problem. Tutoplast^®^ Fascia Lata is a safe option for these patients.

## Introduction

Early allograft dysfunction is a complication that occurs after renal transplantation. It might be due to different causes like kinking or obstruction of the vessels and the ureter. Renal allograft compartment syndrome (RACS) is an infrequent but endangering entity that can lead to early allograft dysfunction and loss of graft (1–3). One of the main causes of RACS in pediatric transplantation is the compression of renal parenchyma or renal vessels due to tight abdominal wall closure, which is frequently a consequence of size discrepancy between the donor and the recipient (4). However, adult-sized kidneys provide better results for children than grafts from matched pediatric donors (5). In addition, children are often offered implants bigger than themselves, as is with the case of living donors from a family member (generally one of the parents). It is becoming a more frequent issue to be addressed since children under 15 kg are exhibiting the safety and the benefit of early renal transplantation (6).

Currently, the best option for chronic kidney disease (CKD) in pediatric patients is renal transplantation. As previously stated, as it is safe even for very small children, renal transplantation should be carried out at the earliest possible time. If possible, it should be conducted before the initiation of renal dialysis to avoid the treatment's effects in children (i.e., high mortality rate, cognitive and learning impairment, anemia, bone disease, growth retardation, etc.) (7). In the last years, new immunosuppression and antiviral regimes have improved pediatric transplant outcomes, showing superiority to outcomes of young adults in some studies (8).

However, transplantation of adult-sized kidneys remains a high-risk procedure in children because of a higher rate of surgical complications. This is due to the technical difficulty of this procedure, specifically during the closure of the abdominal wall and the eventual swelling of neighbor organs. Historically, there has been a tendency of intra-abdominal renal transplantation in the pediatric recipient to avoid complications associated with transplanting a larger adult donor kidney into a small child. This approach is not exempted from difficulties in its performance as many child recipients have already undergone previous abdominal surgeries, per-operatory complications, and post-operatory morbidity (9). Nowadays, the extra-peritoneal approach is the standard of pediatric transplantation, even in children <15 kg. In these circumstances, the surgeon might have to face difficulties during abdominal wall closure that could increase the risk of RACS.

We share in this study our experience with Tutoplast^®^ Fascia Lata in abdominal wall closure with the aim to provide the surgically community with one technique to achieve a tension-free closure. Many surgical modifications to overcome this problem have been described, such as meshes and fascial/muscle flaps (3, 4, 10). The literature is, nevertheless, brief and the use of Tutoplast^®^ Fascia Lata for pediatric transplant has not yet been reported at the time we performed our study.

## Methods

All pediatric patients who underwent renal transplantation in our center between 2013 and 2021 were reviewed. Out of 52 patients, 8 patients with Tutoplast^®^ Fascia Lata placed at the time of initial transplantation were identified.

### Surgical Technique

The operation begins with a Gibson incision that starts 2-finger breadths from the right pubic tubercle and can be extended to the costal margin, if necessary. Allografts are placed on the patient's right/left side to facilitate easier access to the major blood vessels. The abdominal wall is opened in layers until the peritoneum is encountered. The peritoneal membrane is medially reflected through blunt dissection to avoid tearing. Blunt and sharp dissection of the right renal fossa is then performed. This exposes the aorta and iliac vessels in the retroperitoneal space. Once adequate exposure is obtained, the renal allograft is prepared on ice at the back-table. Venous and arterial control is attained using tangential clamps.

Afterwards, the renal allograft is placed in an anatomical position in the surgical field, and end-to- side anastomosis is performed in the recipient's iliac vessels; firstly, to the renal vein of the graft to iliac vein of the recipient and, secondly, to renal graft artery to iliac artery of the recipient with running polypropylene suture material.

The allograft is then placed in the retroperitoneum, avoiding any kinking in the vessels or compression on the anastomosis. We performed in every case an ureteroneocystostomy with anti-reflux system to the bladder. This was also performed in augmented bladders. A ureteral Double J stent is placed in every surgery and removed weeks later.

Directly after closing the abdominal wall, a Doppler ultrasound is routinely performed intra-operatively, maintaining the sterile setting until the confirmation of optimal perfusion of the organ and optimal renal artery and vein parameters.

With this routine, we prevent postoperative respiratory compromise and possible postoperatively instabilities such the use of catecholamines, sedation, and assisted ventilation related to delay in the identification of RACS.

Ultrasound examination was always performed with a 1-6 MHz transducer and started with gray scale imaging to gather information about graft position and size. Afterwards, color Doppler and power Doppler imaging was performed. Renal arterial resistive index (RI) between 0.6 and 0.8 were considered normal. If any sign of vascular compromise or hampered blood flow was seen in the Doppler, such as increased RI, reduced general blood flow, or decreased, reversed, or absent diastolic flow in the spectral Doppler, the abdominal wall was re-opened and the graft was revaluated.

In these cases, the Tutoplast^®^ Fascia lata implant was used to perform tension-free fascia closure (Supplementary Figure 1). The graft was sutured to the fascial edges, and after complete closure, another Doppler ultrasound was performed to confirm the optimal renal artery perfusion and venous patency.

The size of the Tutoplast implant used is 60 x 120 mm (6x12 cm).

There are no conflicts of interest as neither authors nor center had been sponsored by Tutoplast^®^.

## Results

Out of the 52 renal transplants, eight patients received a Tutoplast^®^ Fascia Lata graft.

Overall mortality was 1.9% (one patient without Tutoplast graft). None of the patients required dialysis due to transplant failure till date. One patient, who did not need Tutoplast^®^ Fascia Lata assisting the wall abdominal closure, developed stenosis of the arterial anastomosis 1.5 years after transplantation and required arterial stenting.

From the eight patients with Tutoplast^®^ Fascia Lata, three of the renal transplantations were from cadaver donors, with two of them en bloc (Recipients seven and eight). Living donor transplantation was performed in four cases. In addition, there was one case of bilateral auto-transplantation due to renal artery stenosis due to middle aortic syndrome (Recipient number 6 from Table 1).

**Table 1:** The characteristics of donors and recipients.

**Recipient**	**1**	**2**	**3**	**4**	**5**	**6**	**7**	**8**
Recipient age at the transplantation (years)	10	3	15	10	5	9	13	3
Recipient weight (kg)	23	13	86	25	11	32	43	15,3
Recipient size (cm)	117	92	160	115	88	133	153	94
Donor weight (kg)	100	63	110	70	70	32	13	14
Donor size (cm)	190	189	169	175	178	133	91	91
Cadaver/Living Donor	LD	LD	LD	CD	LD	Auto	CD enbloc	CD enbloc
Last Creatinine value (mg/dl)	0,8	0,3	0,8	0,8	0,2	0,4	0,7	0,4
Time of follow up (months)	42	54	9	29	110	14	9	1
Sex	Male	Male	Female	Male	Female	Female	Female	Female

None of the patients presented any complication of either short or long term that was derived from the abdominal closure with Tutoplast^®^ Fascia Lata.

Mean follow up was 17.8 months.

The characteristics of donors and recipients are shown in Table 1.

## Discussion

We described our experience in the use of Tutoplast^®^ fascia lata for abdominal wall closure in 8 patients belonging to a cohort of 52 renal transplants. Tutoplast^®^ fascia lata is as a commercially available homograft which has been successfully used as human graft tissue and is described in many surgical procedures to be like rhinoplasty and used in the management of stress incontinence (11, 12). The literature describes successful Tutoplast^®^ pericardium utilization in the management of Peyronie's disease (13) and as a patch graft in glaucoma and corneal surgery to cover exposed scleral buckles and oculoplastic surgery (14). However, there is no literature available in the use of Tutoplast^®^ for abdominal wall closure as a method to savage RACS.

Many studies described the use of different materials and techniques to avoid RACS, e.g., prosthetic mesh, polypropylene-assisted mesh hood facial closure fasciotomy, and porcine dermal collagen (1, 3, 4, 15), but literature in this topic is brief, especially concerning pediatric renal transplantation. Techniques, such as intraperitonealization or subcutaneous placement of the allograft, should be avoided and only reserved for extreme cases when the use of other techniques is not possible. Some of the concerns regarding prosthetic mesh, porcine collagen, and materials such as Tutoplast^®^ are the increase of infection and difficulties regarding biopsy or ultrasound of the graft. Several studies have already described their experience with such materials, confirming its safety and the perfect monitoring of the transplanted kidney (3). In addition, a group has described the use in children of porcine dermal collagen (10, 16).

On the other hand, RACS is a rarely seen phenomenon in renal transplantations. In fact, literature described it with a frequency of only 2% (17). The true frequency of RACS is believed to be underestimated in literature as it might be overlooked and/or misdiagnosed as renal vein thrombosis, acute tubular necrosis, or delayed graft function (18). Known data was derived almost exclusively from adult recipient population, and, even then, the pathogenesis is not yet fully understood. It is important to address that restricted volume of the recipient pelvic cavity, such as in small infants, together with size discrepancy between the recipient pelvic cavity space and the donor adult kidney may lead to RACS.

It is also known that contrary to what was believed until the late 1980s, renal transplantation is the best therapy approach for infants and small children. Qvist and colleagues have shown excellent long-term results in 51 children aged younger than 5 years who underwent renal transplantation (19). We have previously published our experience and long term follow up in 132 patients who have undergone pediatric transplantation (20) and found that chronic rejection was the leading cause of graft loss. In the series of Qvist, most of the recipients received adult size allografts. These allografts came from a living related donor in more than one third of the cases. They postulate that tubular and glomerular findings may be the result of chronic graft ischemia when adult size kidneys are transplanted in small children. The higher the disparity in sizes between the donor and the recipient, the more severe the renal ischemia secondary to chronic hypoperfusion tends to be (21). We must take into account that most of the patients who underwent transplantation before 2 years of age have living related donors, which makes the difference between donor/recipient size obvious. Salvatierra et al. (21) demonstrated that in maintaining optimal intravascular volume during the first 6 months after transplantation, a considerable reduction (26%) in the renal mass follows. This is what they believe happens as a consequence of chronic hypoperfusion of the graft. They showed that preserving long term optimum intravascular volume might be more important than immunologic risk factors for the graft survival.

As infants have currently the lowest rate of graft loss after the first year and the longest adjusted allograft survival half-life time (22), we should aim to reduce non immunologic risk factors with the purpose to enhance long term graft survival. Acknowledging the risk of RACS in small recipients and bringing awareness about it are important points to focus along with the surgeons' efforts in guaranteeing to provide the best care for this group of patients who are especially vulnerable to this complication.

We believe that with our routine immediate postoperative ultrasound in pediatric transplantation, we developed a good system do diagnose RACS at an early stage, before it can lead to thrombosis of the renal graft vein and subsequent loss of transplant.

By performing early ultrasound Doppler examination, we can rule out problems, such as acute tubular necrosis and venous thrombosis, that might lead to similar Doppler ultrasound results at a later time point of examination.

In our cohort, (8/52) 15.4% of patients were provided with the Tutoplast^®^ fascia lata graft. This seems to be a high number at first sight, but might be due to the fact that in pediatric transplantations, special limitations are more frequent. Since we did not see any complications, such as graft loss, immune reaction toward the Tutoplast^®^ Fascia Lata graft, wound infections, or healing problems, we postulate that this method is a safe option for pediatric patients to prevent bigger complications and/or graft loss due to RACS.

## Conclusions

Restricted volume of the recipient pelvic cavity and the size discrepancy between the recipient pelvic cavity space and the donor adult kidney may lead to RACS.

We described our experience in the use of Tutoplast^®^ Fascia Lata for abdominal wall closure in 8 patients belonging to a cohort of 52 renal transplants. In these 8 patients, no complication has been seen related to the use of Tutoplast^®^ Fascia Lata.

Other situation occurring more infrequently, such as en bloc or auto-transplantation, are prone to suffer the same problem. Tutoplast^®^ Fascia Lata is a safe option for these patients.

The literature regarding RACS and in children is scarce. Nevertheless, this complication can lead to early loss of the graft. Therefore, we aim to provide our insight in this topic and believe that the results are successful despite having a small cohort.

There is a need for more studies regarding this important topic to achieve the best possible results in pediatric renal transplantation.

## Limitations

We acknowledge as limitation the small cohort of patients and the short follow up of those who recently underwent renal transplantation. Also, the cost of the Tutoplast^®^ Fascia Lata could be a limitation in the reproducibility of this technique in other institutions.

## Data Availability Statement

The original contributions presented in the study are included in the article/Supplementary Material, further inquiries can be directed to the corresponding author/s.

## Ethics Statement

The studies involving human participants were reviewed and approved by Charité Universitätsmedizin Berlin Ethikkomission Campus Virchow Klinikum Antrag EA2/026/22. Written informed consent to participate in this study was provided by the participants' legal guardian/next of kin.

## Author Contributions

BBa and AL: design, data collection, and writing of the article. TG: photographic material and revision of the article. DM: providing data and revision. BBe: data collection and analysis. All authors contributed to the article and approved the submitted version.

## Conflict of Interest

The authors declare that the research was conducted in the absence of any commercial or financial relationships that could be construed as a potential conflict of interest.

## Publisher's Note

All claims expressed in this article are solely those of the authors and do not necessarily represent those of their affiliated organizations, or those of the publisher, the editors and the reviewers. Any product that may be evaluated in this article, or claim that may be made by its manufacturer, is not guaranteed or endorsed by the publisher.

## Supplementary Material

Supplementary Figure 1 Placement of Tutoplast^®^ Fascia Lata graft *in situ*.
