# Supplementary figures and images for: Introducing a New Technique for Fascial Closure to Avoid Renal Allograft Compartment Syndrome in Pediatric Recipients: The Use of Tutoplast® Fascia Lata
Source: Front Surg. 2022 May 6;9:840055. doi: 10.3389/fsurg.2022.840055 (PMC9120621; doi:10.3389/fsurg.2022.840055)

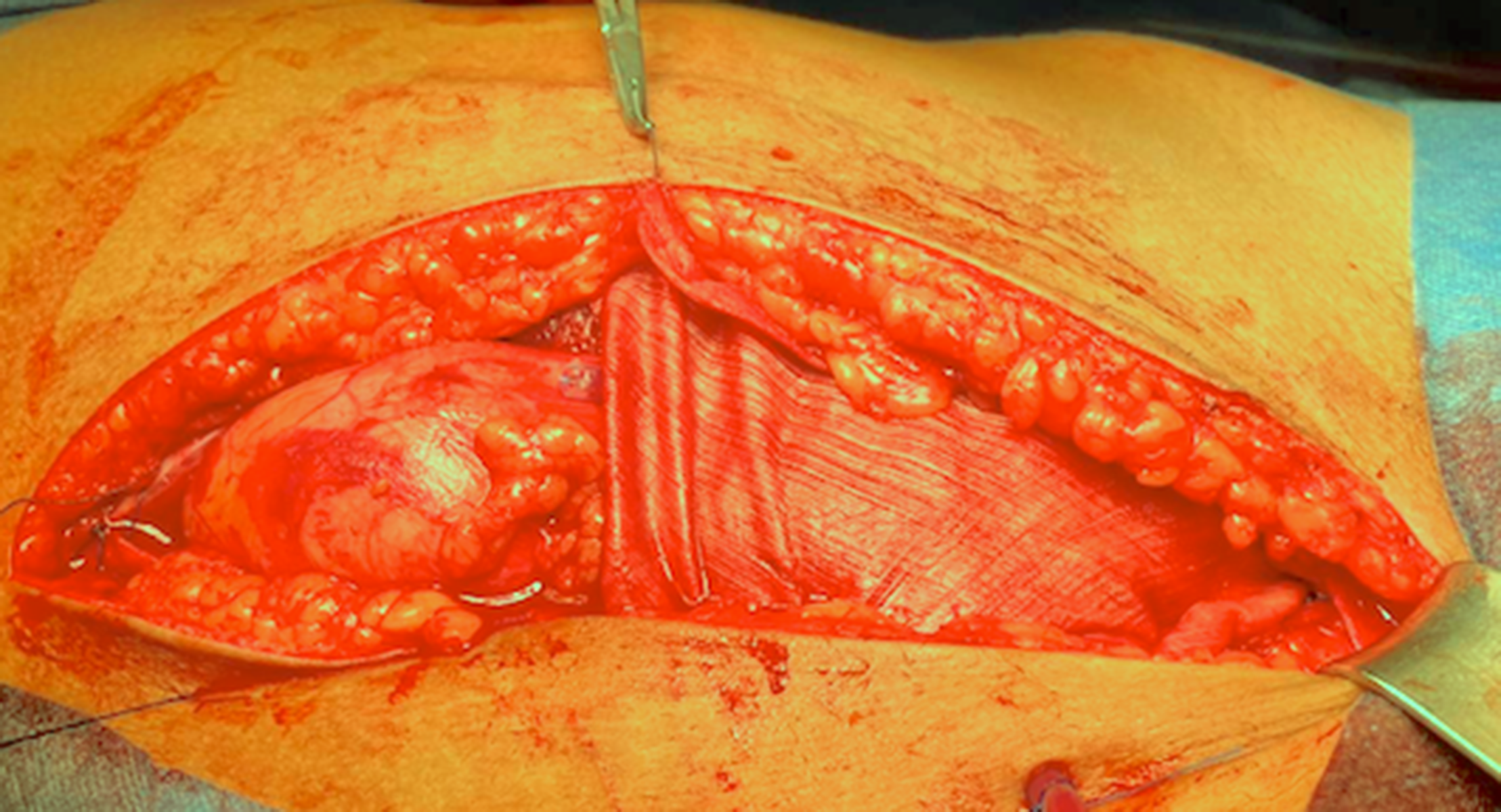

Supplement: Supplementary Figure 1 — Placement of Tutoplast® Fascia Lata graft in situ. [file Image_1.PNG]
